# Palatinose^TM^ (Isomaltulose) and Prebiotic Inulin-Type Fructans Have Beneficial Effects on Glycemic Response and Gut Microbiota Composition in Healthy Volunteers—A Real-Life, Retrospective Study of a Cohort That Participated in a Digital Nutrition Program

**DOI:** 10.3389/fnut.2022.829933

**Published:** 2022-03-07

**Authors:** Anna Kordowski, Axel Künstner, Lisa Schweitzer, Stephan Theis, Torsten Schröder, Hauke Busch, Christian Sina, Martin Smollich

**Affiliations:** ^1^Institute of Nutritional Medicine, University Hospital of Schleswig-Holstein, Campus Lübeck and University of Lübeck, Lübeck, Germany; ^2^Group of Medical Systems Biology, Lübeck Institute of Experimental Dermatology, University of Lübeck, Lübeck, Germany; ^3^BENEO-Institute, BENEO GmbH, Obrigheim, Germany; ^4^Perfood GmbH, Lübeck, Germany

**Keywords:** palatinose™, isomaltulose, inulin-type fructans, prebiotics, personalized nutrition, continuous glucose measurement, citizen science, microbiota

## Abstract

It is well-appreciated that the diet is a crucial tool to counteract cardiometabolic disturbances due to its impact on blood glucose concentration and gut microbiome. This retrospective analysis aimed to examine whether the inclusion of isomaltulose and prebiotic inulin-type fructans (ITF) into the habitual diet has an impact on glycemic control and gut microbiota. Furthermore, we examined interindividual differences in glycemic response to sugar replacement with isomaltulose. We retrospectively analyzed data of 117 individuals who participated in a digital nutrition program including a 14-day continuous glucose measurement. Participants underwent six test days with sweetened drinks (isomaltulose vs. sucrose) consumed with their usual breakfasts and lunches. Dinner was supplemented with ITF for 11 days. Postprandial glycemia and 24 h-glycemic variability were determined following test meals and days, respectively. Fecal microbiota was analyzed by 16S rRNA sequencing before and after test phase. Meals with isomaltulose-sweetened drinks compared to meals with sucrose-sweetened drinks induced lower postprandial glycemia. Moreover, glucose oscillations over 24 h were lower on isomaltulose when compared to sucrose test days and improved further during ITF supplementation. Furthermore, ITF modulated gut microbiota composition beneficially. Responder analysis revealed that 72% of participants benefited from the sugar replacement with isomaltulose and that their gut microbiota differed from the low responders. Taken together, the incorporation of isomaltulose and ITF into the habitual diet was shown to be an effective strategy to improve glucose control and beneficially modulate gut microbiota, and thereby aid to maintain metabolic health. Data indicate interindividual differences in glycemic response to ingredients and suggest that gut microbiota might be somehow related to it.

## Introduction

There is a high prevalence of impaired glucose tolerance and prediabetes in the general population (1, 2). In turn, hyperglycemia as well as a high glycemic variability are important risk factors for cardiometabolic diseases and type 2 diabetes (T2D) (3–5). Consequently, there is an urgent demand for effective strategies to prevent elevated blood glucose concentrations. A central element in those strategies are dietary modifications since individual diet represents a fundamental determinant of blood glucose regulation (6, 7).

A systematic reduction of the dietary glycemic index (GI) could be an important approach to improve postprandial glucose response. It has been shown previously that replacing rapidly digestible, high-GI foods by slowly digestible, low-GI foods contributes to reduced postprandial glycemia and insulinemia and lowers glycemic variability (8–11). Moreover, there is sufficient evidence that a frequent consumption of high-GI foods is associated with an increased risk of cardiometabolic diseases when compared to a diet rich in low-GI foods (12, 13).

However, regardless of the established benefits of a low-GI diet for the general population, the glycemic response to identical foods is subject to considerable interindividual variability (14–16). Therefore, in order to meet personalized dietary needs, individualized recommendations rather than general dietary advice could be useful to obtain the greatest possible improvements in daylong glycemia (17, 18). Zeevi et al. (18) were the first to systematically examine potential determinants of the high interindividual variability of postprandial glucose response in an expressive study population. Apart from factors like anthropometrics, lifestyle behaviors and clinical parameters, the gut microbiota has been identified as an important predictor of individual glycemic response. Furthermore, applying personalized dietary interventions resulted in lower postprandial glycemia and consistent changes in gut microbiota (18). Besides Zeevi and colleagues, also other researchers demonstrated a strong link between gut microbiota and glycemia or associated outcomes (19–21), and hence, the targeted modulation of the microbiota seems to be a further suitable approach to improve blood glucose regulation.

An established way to achieve a beneficial shift in microbiota is the supplementation with prebiotics (22, 23). This approach would furthermore increase the daily fiber intake and thus help to fill the existing “fiber gap” (24), which is, given the essential role of dietary fibers in maintaining human health, of high interest (25).

For this retrospective analysis, data have been drawn from a general population analysis originally performed by Perfood GmbH. The initial research aim was to include isomaltulose and prebiotic inulin-type fructans as a part of freely chosen test meals in order to confirm the quality of the methodology used in the digital nutrition program.

Isomaltulose (tradename Palatinose™, ISO) is a fully digestible, low-GI sucrose isomer naturally occurring in honey and sugar cane juice (26). Since the α-1,6-glycosidic bond between fructose and glucose molecules is stronger compared to e.g., that of sucrose, its digestion is lower, which leads to a lower and more sustained postprandial glycemia accompanied by a lower glycemic variability (26).

Inulin-type fructans (ITF) are prebiotic dietary fibers naturally occurring in plants, such as onions, artichokes, chicory or garlic (23). Through the selective fermentation of ITF by gut bacteria, especially *Bifidobacteria*, they mediate health-promoting effects (23).

In this retrospective analysis, we examined whether the intervention with two functional food ingredients with established health benefits, that are slowly digestible, low-GI ISO, and prebiotic ITF, is an effective strategy to improve blood glucose control and modify gut microbiota in a real-life setting. Furthermore, we evaluated differences in the individual responses and aimed to identify participants who particularly benefit from the ingredients and whether these could be distinguished by their underlying gut microbiota composition.

## Methods

### Initial Intention of the Investigation

The initial investigation was a general population analysis conducted by Perfood GmbH (Germany), a company offering a digital nutrition program aiming to control body weight by applying personalized nutrition recommendations. More specifically, interested persons can run a 2-week test phase which includes freely chosen test meals. Individual responses to test meals using state-of-the-art methodology, i.e., continuous glucose monitoring (CGM) and gut microbiome RNA sequencing, are analyzed and personalized, tailored nutrition recommendations derived.

To prove the quality of the methodology and software used, Perfood GmbH included the commercially available functional ingredients, Palatinose™ and Orafti®Synergy1 provided by BENEO GmbH, in their digital nutrition program. Since this was part of the evaluation of test meals, no ethical committee approval was required.

### Design

In total, 120 volunteers from the general population were recruited by Perfood GmbH using different social media channels. In total, 117 participants completed the sensor-assisted test phase; 231 gut microbiota samples were collected. All participants gave their informed consent to use their anonymized data for scientific purposes. The Institute of Nutritional Medicine at the University of Lübeck was asked to evaluate the data regarding human metabolism in order to subsequently prepare prospective clinical trials.

**Figure 1:**
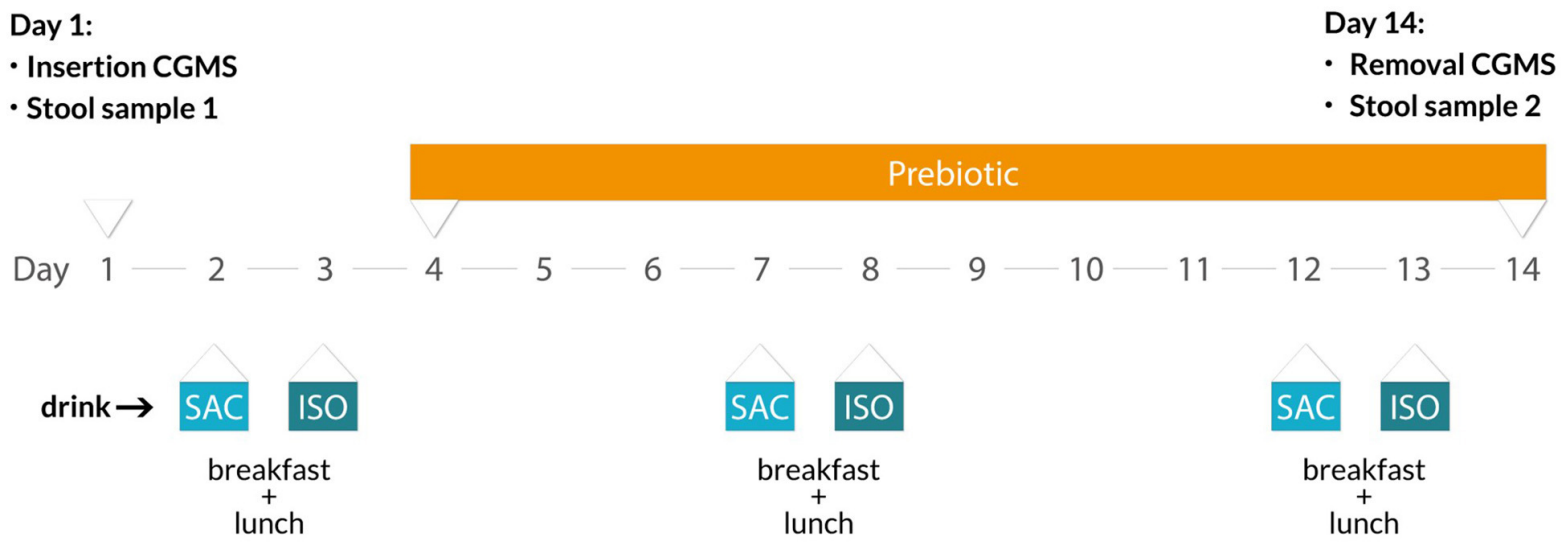
Study design. Drinks containing the sucrose (SAC) and isomaltulose (ISO) were consumed with breakfasts and lunches on days 2, 7, 12 for SAC and 3, 8, 13 for ISO. During the study, drink composition was double-blinded. Prebiotic inulin-type fructans were supplemented from day 4 until day 14. Continuous glucose monitoring sensor (CGMS) was inserted on day 1 and removed on day 14. The stool samples were collected on days 1 and 14.

The participants took part in a 14-day test phase throughout which glucose response was tracked continuously. Therefore, participants were instructed to insert the glucose monitoring sensor on day 1 and remove it on day 14. During the test phase, participants were allowed to freely choose their meals. Besides this, on selective days, they were asked to consume test drinks containing either isomaltulose (ISO; Palatinose™, BENEO GmbH, Germany) or sucrose (saccharose, SAC; Südzucker AG, Germany) together with their habitual breakfast and lunch. More specifically, SAC drinks were consumed on days 2, 7, and 12, whereas ISO drinks were consumed on days 3, 8, and 13 (Figure 1). Therefore, instant powders containing 30 g of available carbohydrates were dissolved in 250 ml water. The participants were allowed to have their habitual breakfast and lunch without further instructions; additionally, they were asked to eat comparable meals on all test days. Both, the participants, and the data analyst were blinded, with un-blinding taking place after data analysis. From day 4, participants were asked to supplement their daily diet with 10 g prebiotic ITF (i.e., an oligofructose-enriched inulin, Orafti®Synergy1, BENEO GmbH, Germany) which was consumed together with their habitual dinner.

At the beginning and end of the test phase, participants were instructed to take stool samples and to complete a medical questionnaire. Relevant lifestyle habits such as food intake or sport activities were tracked with the MillionFriends App (www.millionfriends.de).

### Glucose Measurements

Continuous glucose monitoring (CGM) was performed daily throughout the test phase using the Abbott FreeStyle Libre system (Abbott GmbH, Germany). Postprandial glycemic response following the ingestion of test meals (i.e., breakfast and lunch with SAC and ISO drinks) was expressed by the AUCi (incremental area under the curve). The AUCi was calculated by the trapezoidal rule using the glucose values determined during 120 min after the meals have been logged by the participants into the MillionFriends App. For further characterization of glycemic control, additional parameters such as glucose peak (Cmax), glucose nadir (Cmin) as well as the times at which peak (Tmax) and nadir (Tmin) occur were determined for 180 min post-meal. Of note, participants were instructed to have a minimum interval of 2 h between two meals and/or a meal and any physical activity. Mean amplitude of glucose excursion (MAGE) over 24 h was calculated using the R package gluvarpro version 2.0.0 in order to compare the glycemic variability between consecutive SAC and ISO test days at the beginning (day 2 vs. 3), the middle (day 7 vs. 8), and end (day 12 vs. 13) of the test phase. Higher MAGE values reflect greater glycemic variability indicating a greater amplitude of glycemic fluctuations over a 24 h period. Moreover, the glycemic variability on test days at the beginning (i.e., days 2 and 3) was compared with that on a control day. Since at baseline, on day 1, CGM calibration took place, day 4 was used which was a day without test drink consumption and the first day of ITF supplementation.

To characterize glycemic condition of the participants, detailed glucose profiles were used to calculate fasting glucose concentrations, average glucose concentrations as well as glycated hemoglobin (HbA1c). Fasting glucose was determined as the baseline glucose concentration using a proprietary algorithm developed by Perfood GmbH. Briefly, the baseline is predicted on daily individual 24 h glucose profiles combined with data from individual medical records. HbA1c was calculated following Temsch et al. by multiplying the average glucose concentration of the complete test phase with 0.03 and adding 2.6 (27). Of note, HbA1c as calculated in this study is not of the same quality as HbA1c measured in standardized laboratories.

### Fecal Microbiota Analysis

The fecal microbiota composition was analyzed via 16S ribosomal DNA sequencing. For this purpose, participants sent in two stool samples taken on days 1 and 14 of the test phase (Figure 1). The samples were stored in PSP Spin Stool DNA plus kit provided by Invitek Molecular (Germany) with 8 ml DNA stabilizer. After arrival, samples were stored at −80°C. Extraction, isolation, and sequencing was performed at Microsynth AG (Switzerland). Postal transfer to the lab was performed at room temperature. After sequencing, microbiome data were imported as “fastq files” and preprocessed using an in-house pipeline comprising merging of paired reads, quality filtering, chimera removal using vsearch (v2.8.0) and taxonomic assignment against NCBI 16s databases using malt (v0.4.1). Preprocessed files were converted into biom format and analyzed using R packages phyloseq (1.30.0) and vegan (2.5–6). Alpha diversity was estimated using Chao1 and Shannon index, beta diversity was estimated using Bray-Curtis dissimilarity and principal coordinate analysis (PCoA) was used to visualize the results. Sequencing data used for this study were submitted to the European Nucleotide Archive (ENA) and are available under accession number PRJEB49980.

### Responder Analysis

Since participants differed in the extent of their response to the ISO and SAC test drinks, a responder analysis has been performed. For this, the difference in MAGE between days 3 (ISO) and 2 (SAC) was considered as main readout. With a minimum difference of 7.5%, individuals were considered as good responders. Consequently, a participant was classified as a good responder with a ratio above 1.075 and as a low responder with a ratio below 0.925. To classify responders, only participants that ate all three sets of required breakfasts (days 2 vs. 3, 7 vs. 8, 12 vs. 13; *n* = 70) were included. In order to compare the composition of the fecal microbiome between good and low responders, stool sample 1 was used.

### Statistical Analysis

Statistical analyses were performed using R (v3.6.2). Statistical tests were performed using non-parametric tests (Wilcoxon–tests and Kruskal–Wallis test). If necessary, tests were performed in a paired test framework. Statistical significances were reported using uncorrected *p*-values or *p*-values that were corrected for multiple testing using Benjamini–Hochberg correction (denoted as q-values). Differences in beta diversity were assessed by permutational multivariate analysis of variance (PERMANOVA) using distance matrices with 9,999 permutations to calculate significance values.

## Results

### Cohort Description

In total, 117 participants completed the sensor-assisted test phase; 231 microbiota samples were collected. The study cohort consisted of 73.5% female (*n* = 86) and 26.5% male (*n* = 31). Mean age was 42.9 years. Mean weight was 84.9 kg with a mean BMI of 28.7 kg/m^2^ (Table 1).

**Table 1:** Baseline characteristics of the test cohort as well as a randomly assigned reference cohort^a^, ^b^.

**Variable**	**Test cohort**	**Reference cohort**
Participants, *n*	117	117
Female, *n*	86	88
Age, y	42.9 ± 11.3	42.4 ± 11.9
Body weight, kg	84.9 ± 20.5*	80.0 ± 23.6
Body weight 1 y before, kg	84.5 ± 19.6*	77.8 ± 21.2
BMI, kg/m^2^	28.7 ± 6.7*	26.9 ± 6.8
Nutritional status, %		
Underweight	0.0	1.2
Normal weight	29.9	39.3
Overweight or obesity	69.3	58.2
Weekly activity, h	9.4 ± 6.2	8.9 ± 5.8
Energy intake, kcal/d	2,168 ± 468	2,047 ± 389
Fat, energy%	36.0 ± 6.1	37.3 ± 5.1
Carbohydrates, energy%	45.4 ± 7.0	44.6 ± 6.5
Protein, energy%	16.2 ± 3.3	16.4 ± 3.6
Fiber, energy%	2.7 ± 0.7	2.0 ± 0.7
Fiber, g/d	28.6 ± 7.8	21.4 ± 4.8
Hba1c_cal_, %	5.56 ± 0.32*	5.28 ± 0.27
Average glucose, mg/dl	98.7 ± 10.3*	89.3 ± 8.7
Fasting glucose, mg/dl	89.6 ± 10.8*	82.7 ±10.3

a *Data presented as mean ± SD*.

b BMI, body mass index; Hba1c_cal_, calculated value of glycated hemoglobin. Significant differences between cohorts were assessed using Wilcoxon test: *p < 0.05.

In order to examine whether the test cohort represents people usually participating in the digital nutrition program from Perfood without the inclusion of functional ingredients, a cohort was randomly assigned from the MillionFriends database (in the following called reference cohort). For this, an equal subject number (*n* = 117) was randomly selected without any pre-filtering for any covariate (e.g., age, weight, or gender; Table 1).

The reference cohort consisted of 75.5% (*n* = 88) female and 24.8% (*n* = 29) male. The mean age of the reference cohort was 42.4 years which did not differ from the test cohort. The participants of the test cohort when compared to the reference cohort reported a higher weight (84.9 vs. 77.8 kg) but a lower mean weight increase (0.45 vs. 2.18 kg) over the 12 months before the test phase. The body mass index (BMI) was 1.8 kg/m^2^ higher for participants of the test cohort when compared to the reference. The distribution of the body weight in the categories underweight, normal weight, overweight, and obesity WHO grade I° to WHO III° did not differ between the test and the reference cohort. Of the test cohort, 34.2% were overweight and 35.1% were obese. On average the participants in the test cohort were 9.4 h physically active per week (sports, household, garden work), which was in the range of the reference cohort.

Mean daily energy, macronutrient and fiber intake did not differ between test and reference cohorts (Table 1). To avoid any underreporting bias, for mean energy intake calculation, only days were included on which in total >1,200 kcal were logged into the MillionFriends app. On average, participants of the test cohort received 45.4% of daily energy from carbohydrates, 36.0% from fat, and 16.2% from proteins. Mean daily fiber intake was 28.6 and 21.4 g in the test and reference cohort. Difference between cohorts can be explained by the additional fiber intake in the test cohort which is included in the analysis. Thus, data show that only after supplementing the diet with dietary fiber, the recommended intake level of 30 g/d were almost reached. Taken together, nutrient parameters indicate similar dietary habits of test cohort when compared to the reference cohort.

The mean of the continuous glucose values obtained during the test phases, i.e., fasting glucose, average glucose and the calculated HbA1c were higher in the test cohort as compared to the reference cohort (Table 1).

The breakfasts and lunches at which the participants had the ISO and SAC drinks were, except for the drinks, freely chosen by the participants. Therefore, energy and macronutrient intakes of the meals on days 2, 7, 12 for SAC and on days 3, 8, 13 for ISO were analyzed and compared. This was performed to rule out these factors as confounders when analyzing glycemic reactions. For the analysis of nutrient composition of the breakfasts and lunches, only participants who consumed all six breakfasts and lunches with the test drinks were included. Accordingly, analysis for breakfast and lunch was done in 70 and 65 participants, respectively.

Neither the energy nor the amount of carbohydrates, proteins, or fat differed between test meals with SAC or ISO drinks (Supplementary Figure 1). This indicates that the meals can be used for a proper comparison of glycemic reactions to the test drinks.

In addition, the daily energy and macronutrient intakes were compared between consecutive SAC and ISO test days at the beginning (days 2 and 3), the middle (days 7 and 8), and the end (days 12 and 13). The analysis revealed that daily energy intake as well as the amount of carbohydrates, proteins, and fat were similar when comparing SAC and ISO test days (data not shown).

### Glucose Response—Comparing the Effects of ISO and SAC

**Figure 2:**
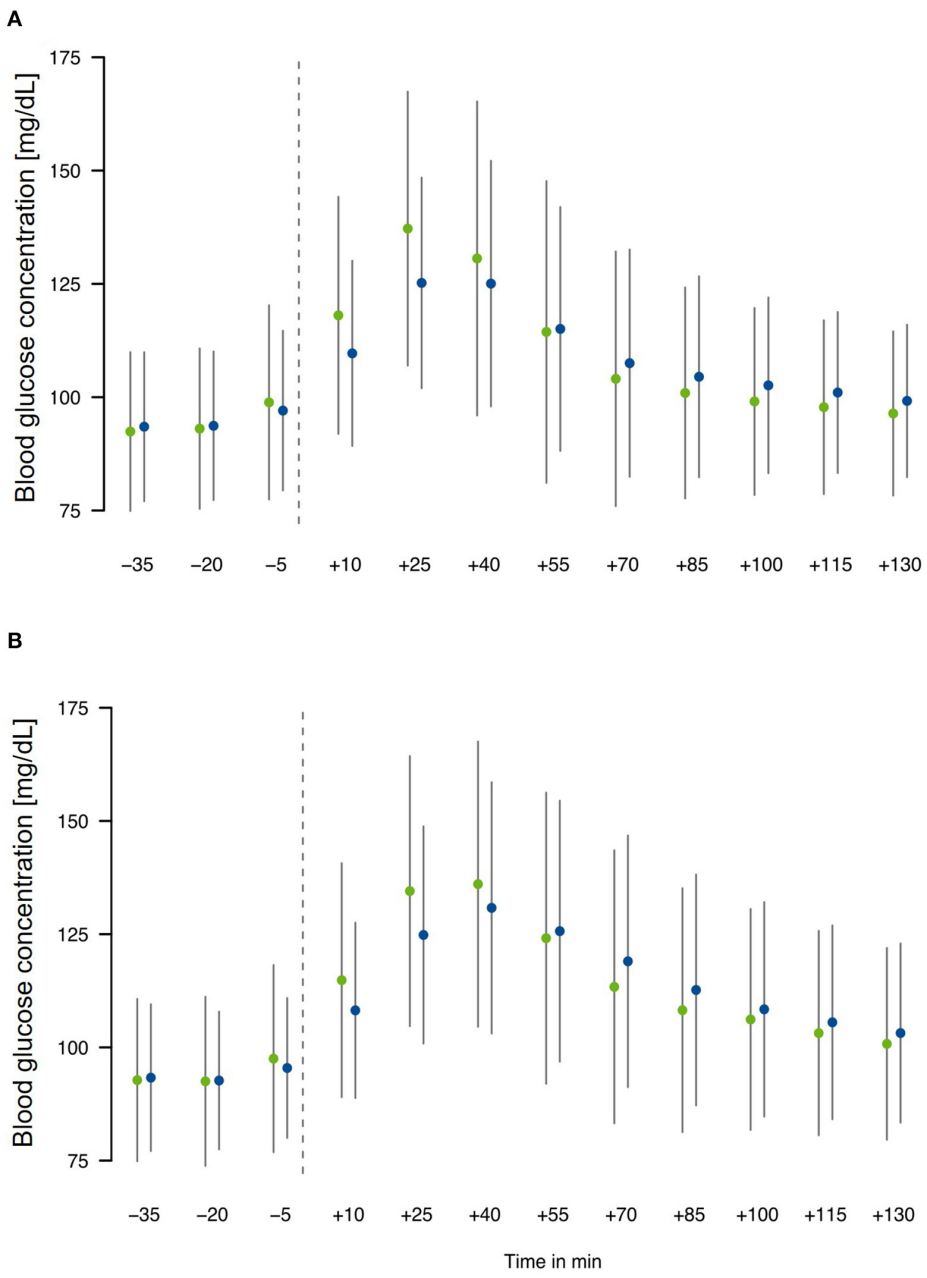
Pooled postprandial glucose response following the ingestion of test meals: **(A)** breakfast and **(B)** lunch with either the SAC drink (green) or the ISO drink (blue) at single time points (*n* = 319 and *n* = 318 for breakfast with SAC and ISO; *n* = 301 and *n* = 296 for lunch with SAC and ISO).

To address the question whether meals consumed with ISO induced a lower postprandial glucose response, the absolute glucose values from 35 min before meal intake (gray line) to 130 min after meal intake were pooled (Figure 2). For this again *n* = 637 breakfasts (SAC *n* = 319, ISO *n* = 318) and *n* = 597 lunches (SAC *n* = 301, ISO *n* = 296) were considered. For both meals, breakfast and lunch, mean glucose values after meal intake were lower with ISO compared to SAC in the first hour, while mean glucose remained higher with ISO in the second hour post-prandially (Figure 2).

Two-hour glycemic response (i.e., AUCi_2h_) following the breakfast with ISO was significantly lower when compared to the breakfast with SAC in the middle (day 7 vs. 8) and at the end (day 12 vs. 13) of the test phase (Table 2). Glucose peak (Cmax) was consistently lower after breakfast and lunch with ISO drinks as compared to meals with SAC drinks, albeit this was statistically significant for breakfast at the beginning of the test phase only, i.e., on day 3 vs. 2. Moreover, glucose peak occurred later when ISO was added to the meals as compared to SAC which was significant for the breakfasts at the beginning of the test phase (i.e., days 2 vs. 3) as well as for all lunches (Table 2). With respect to the glucose nadir (Cmin), in comparison to the addition of SAC, significant higher values were observed for the breakfast and lunch with ISO drinks at the beginning of the test phase (i.e., days 3 vs. 2; Table 2). Thus, data confirm higher minimal glucose concentrations and thereby indicating a prolonged glucose supply. Regarding the time when glucose nadir occurred, the only difference was observed following the lunch on days 7 and 8, showing a later Cmin with SAC in comparison to ISO (Table 2).

**Table 2:** Glycemic response to the test meals^a^.

	**Day**	**iAUC_**2H**_ (mmol/L)**	**Cmax (mmol/L)**	**Cmin (mmol/L)**	**Tmax (h)**	**Tmin (h)**
SAC Breakfast	2	196.7 ± 114.71	146.67 ± 32.41	80.24 ± 16.78	0.71 ± 0.23	1.71 ± 0.89
ISO Breakfast	3	180.73 ± 117.01	136.49 ± 26.76	85.29 ± 12.73	0.93 ± 0.45	1.68 ± 1.08
ΔBreakfast_(ISO−SAC)_		*−15.97*	−10.18^*^	5.05^**^	0.22^**^	*−0.03*
SAC Breakfast	7	201.49 ± 126.76	151.49 ± 29.87	85.14 ± 14.27	0.79 ± 0.39	1.91 ± 0.92
ISO Breakfast	8	165.92 ± 98.02	136.01 ± 26.74	87.65 ± 16.48	0.96 ± 0.54	1.87 ± 0.99
ΔBreakfast_(ISO−SAC)_		−35.57^*^	***–**15.48*	*2.51*	*0.17*	***–**0.04*
SAC Breakfast	12	205.05 ± 127.83	147.1 ± 29.62	85.85 ± 14.92	0.93 ± 0.56	1.86 ± 0.95
ISO Breakfast	13	168.42 ± 88.37	135.87 ± 23.49	86.49 ± 12.97	1.05 ± 0.54	1.76 ± 0.98
ΔBreakfast_(ISO−SAC)_		−36.63^**^	***–**11.23*	*0.64*	*0.12*	***–**0.10*
SAC Lunch	2	206.05 ± 101.19	145.75 ± 25.30	80.83 ± 13.95	0.85 ± 0.41	1.73 ± 1.07
ISO Lunch	3	224.32 ± 134.84	140.85 ± 28.54	87.08 ± 13.93	1.02 ± 0.43	1.58 ± 1.11
ΔLunch _(ISO−SAC)_		*18.27*	*-4.90*	6.25^**^	0.17^*^	***–**0.15*
SAC Lunch	7	228.04 ± 154.42	150.89 ± 29.05	89.72 ± 14.46	0.85 ± 0.45	1.83 ± 0.95
ISO Lunch	8	188.58 ± 103.80	137.72 ± 25.23	87.33 ± 13.75	1.14 ± 0.65	1.52 ± 1.13
ΔLunch _(ISO−SAC)_		***–**39.46*	***–**13.17*	***–**2.39*	0.29^*^	−0.31^*^
SAC Lunch	12	245.23 ± 154.02	151.79 ± 31.64	87.65 ± 14.28	0.87 ± 0.35	1.44 ± 1.10
ISO Lunch	13	209.8 ± 110.52	139.06 ± 27.03	87.51 ± 15.22	1.07 ± 0.53	1.55 ± 1.13
ΔLunch _(ISO−SAC)_		***–**35.43*	***–**12.73*	***–**0.14*	0.20^*^	*0.11*

a *Data presented as mean ± SD. Significant differences between test meals are presented in bold and were assessed using Wilcoxon test*:

** *p < 0.001*,

* *p < 0.05*.

**Figure 3:**
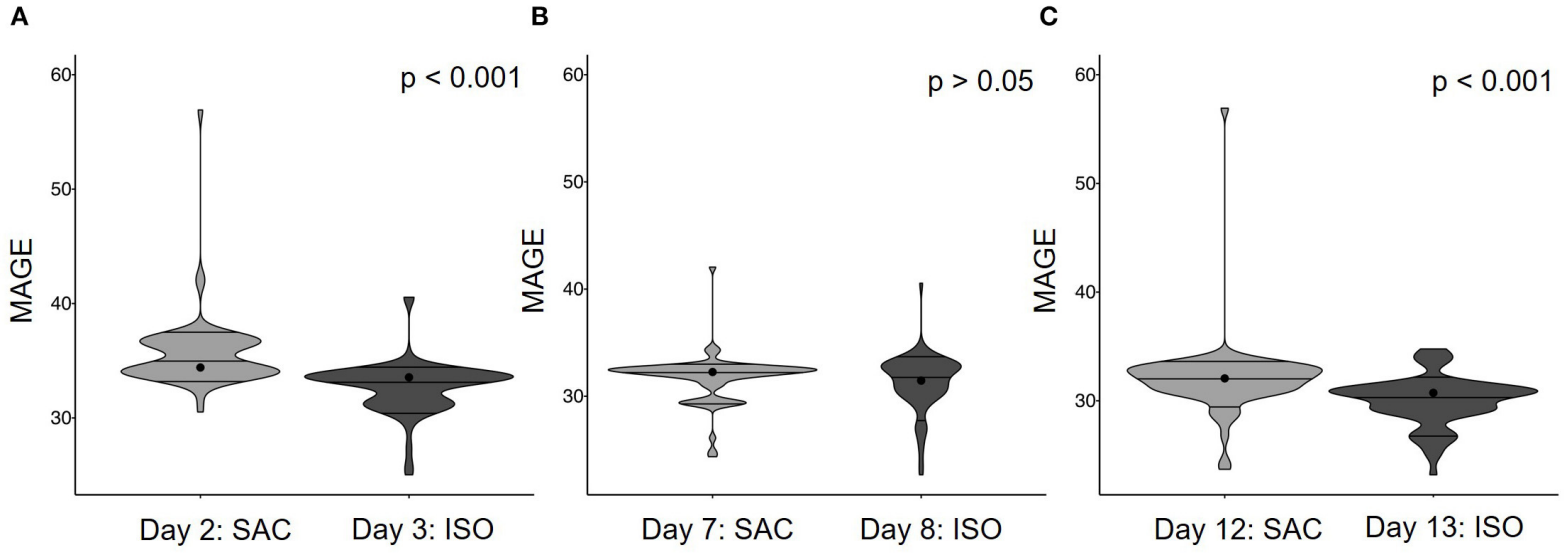
Mean amplitude of glycemic excursions (MAGE) over 24 h following test breakfasts containing SAC or ISO consumed on consecutive test days: **(A)** day 2 vs. 3, **(B)** day 7 vs. 8, **(C)** day 12 vs. 13. Significant differences between test breakfasts were assessed using the Wilcoxon test: *p* < 0.05.

To determine glucose variability, MAGE over 24 h was calculated and compared between consecutive SAC and ISO test days at the beginning (days 2 and 3), the middle (days 7 and 8) and the end (days 12 and 13) of the test phase. Data reveal a significant difference between ISO and SAC days at the beginning and the end of the test phase, clearly indicating lower glucose oscillations on ISO days (Figures 3A,C). On days 7 and 8 no difference was detected (Figure 3B).

The comparison between MAGE on test days at the beginning (i.e., day 2 and 3) and the control day (i.e., day 4) demonstrated a similar glycemic variability on the SAC test day as compared to the control day. In contrast, glycemic variability on the ISO test day was lower compared to both, the SAC test day and the control day (Supplementary Figure 2).

### Glucose Response—Effects of Continuous Prebiotic Supplementation

**Figure 4:**
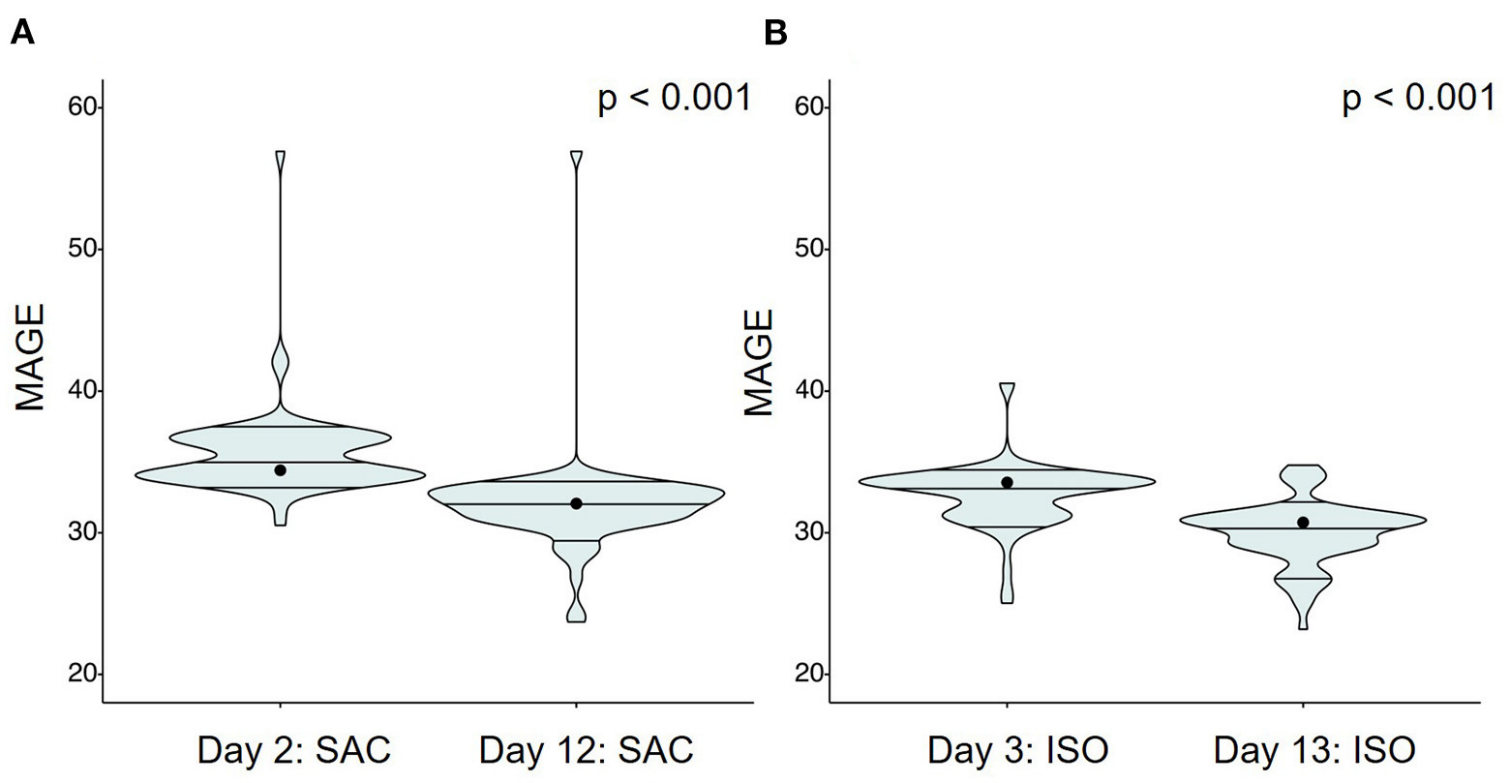
Mean amplitude of glycemic excursions (MAGE) over 24 h following test breakfasts containing **(A)** SAC or **(B)** ISO at the beginning and the end of intervention. Significant differences were assessed using the Wilcoxon test: *p* < 0.05.

In order to analyze potential effects of ITF on glycemic variability, MAGE over 24 h of the ISO and SAC test days at the beginning were compared to those at the end of the test phase after 8–9 days of ITF intake. Accordingly, for ISO, MAGE on day 3 was compared to MAGE on day 13, whereas for SAC, comparison was made between MAGE on day 2 vs. 12. Irrespective of the carbohydrate consumed, a significant decrease in glycemic variability over time, and thus, after ITF supplementation was noted (Figure 4).

### Fecal Microbiota

ITF are accepted prebiotics known to impact intestinal microbiota composition. Accordingly, we performed a comparative analysis of fecal microbiome on day 1 before ITF supplementation and on day 14 after continuous ITF supplementation of *n* = 103 subjects. After 16S gene sequencing and preprocessing, 32.922 ± 16.794 contigs (mean) were reached and represent taxonomic annotation. For comparison a subsampling of 7,400 contigs was performed.

**Figure 5:**
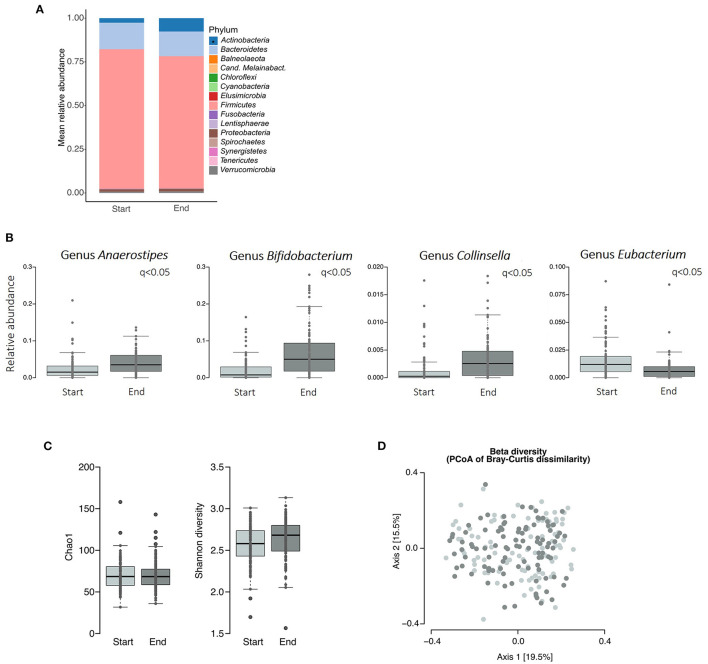
Gut microbiome profiles before and after prebiotic supplementation. **(A)** Relative phylum abundance, **(B)** relative abundance of the genera *Anaerostipes, Bifidobacterium, Collinsella*, and *Eubacterium*, **(C)** alpha diversity, **(D)** Beta diversity (Principal Coordinate Analysis of Bray-Curtis dissimilarity). Significant differences were assessed using Wilcoxon-test and *p*-values were adjusted for multiple testing (*q*-values); asterisk denote significant differences on phylum abundance.

On phylum level, relative abundance of *Actinobacteria* significantly increased over the course of ITF supplementation (Figure 5A). Other phyla and the *Firmicutes*/*Bacteroidetes* ratio did not differ significantly (*q* > 0.05).

Analyzing differences in the relative abundance of genera revealed that *Bifidobacteria, Collinsella*, and *Anaerostipes* increased, and *Eubacterium* decreased over the course of ITF supplementation (*q*-values: *Bifidobacterium q* < 0.001, *Collinsella q* < 0.001, *Anaerostipes q* < 0.05, *Eubacterium q* < 0.001; Figure 5B).

In line with the genera changes, beta diversity changed between the two sample points of the study (PERMANOVA: *p* = 0.0001, *R*^2^ = 0.03067), while alpha diversity remained stable. This clearly indicates a community shift but no change in species numbers after ITF intake (Figures 5C,D).

### Responder Analyses

A cut-off of 7.5% was used to classify good responders and low responders based on the difference in MAGE between SUC test day 2 vs. ISO test day 3. In total, 50 participants were found to be good responders (71% of *n* = 70), whereas 20 were found to be low responders (29% of *n* = 70). None of the participants was classified as a negative responder. A stratification of the different responders regarding age, gender, weight parameters, glucose parameters, and nutritional parameters revealed no differences.

**Figure 6:**
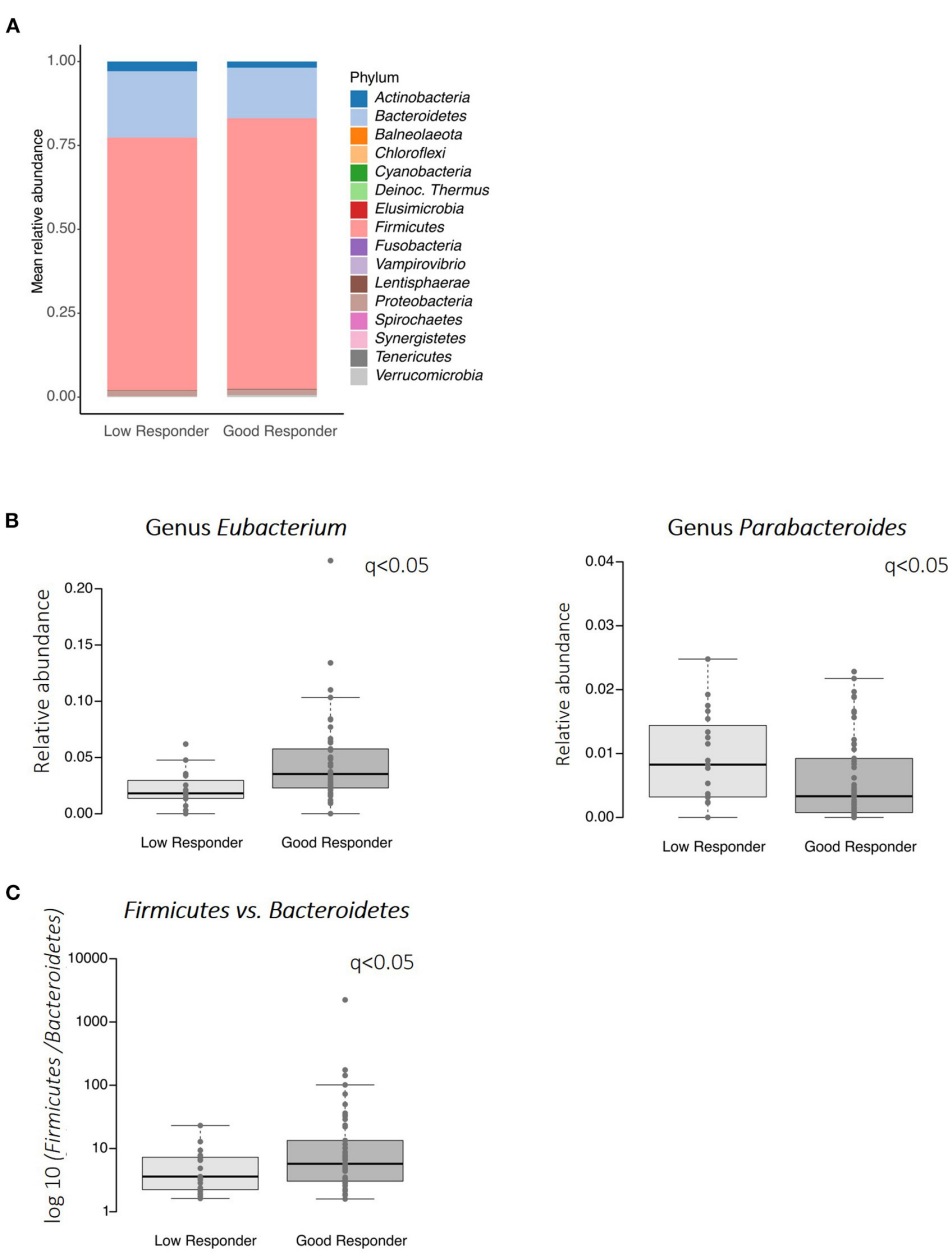
Gut microbiome profiles in good and low responders. **(A)** Relative phylum abundance, **(B)** abundance of the genera *Eubacteria* and *Parabacteroides*, **(C)** the ratio of *Firmicutes* to *Bacteroidetes* (log10). Significant differences were assessed using Wilcoxon test and *p*-values were adjusted for multiple testing (*q*-values).

For comparing the fecal microbiome between the different responders, *n* = 50 and *n* = 19 stool samples were available from good and low responders. After 16S gene sequencing and preprocessing, 31.292 ± 12.133 contigs (mean) were reached and represent taxonomic annotation. For comparison, a subsampling of 9,200 contigs was performed. On phylum level, no difference was detected between good responders and low responders (Figure 6A). However, on genus level significant differences (*q* < 0.05) have been found (Figure 6B). The relative abundance of *Eubacterium* were higher and *Parabacteroides* were lower in good responders as compared to low responders. The ratio between *Firmicutes* and *Bacteroidetes* revealed a trend for a higher value in good responders (Figure 6C, *p* = 0.097). No differences were seen in alpha (Chao1, Shannon) or beta (Bray-Curtis) diversity (data not shown).

## Discussion

Several human intervention studies demonstrated the acute blood glucose-lowering effects of ISO when compared to rapidly available, high-glycemic index carbohydrates (26, 28, 29). Furthermore, prebiotic ITF have been shown to improve postprandial glycemic response (30–32). However, these effects have not been investigated yet in real-life trials. Therefore, to our knowledge, this retrospective study is the first one to explore the physiological effects of ISO and ITF on glycemic response and gut microbiome composition in volunteers from the general population in a habitual, real-world lifestyle setting.

As major findings, the consumption of ISO drinks instead of SUC drinks with usual breakfasts and lunches lowered postprandial glycemia as well as 24-h glycemic variability. Furthermore, glycemic variability improved in the course of the 11-day ITF supplementation. Besides this, continuous supplementation with prebiotic ITF selectively modified gut microbiome composition, characterized by a significant increase in the relative abundance of *Bifidobacteria*. These observations are in line with previous evidence from clinical trials (31, 33–35).

Like different previous studies, we used CGM to determine individual glycemia. This approach facilitates a detailed analysis of glycemic variability, which is of outstanding relevance in diabetes management (36). Recurrent glucose fluctuations contribute to the development and progression of cardio-metabolic disturbances (3), making them even worse than a constantly high glycemia (37–41).

Our findings on glycemic variability, i.e., the acute reduction in MAGE when comparing two subsequent test days as well as the additional reduction in MAGE over time, reflect the different mechanisms by which ISO and ITF influence glycemia.

More specifically, ISO exerts an acute glucose-lowering effect when replacing high-GI carbohydrates as a consequence of the slower hydrolysis and absorption in the small intestine. In contrast, ITF mainly exerts longer-term benefits on glycemia due to its intestinal fermentation.

The present findings on acute MAGE are in line with a 3-day cross-over intervention study in Chinese, demonstrating a reduction in 24-h glycemic variability when sucrose was replaced by isomaltulose (8). Similar findings were observed by an inactivity study conducted by Keller et al. in healthy, active men (42). After only 1 week of physical inactivity and the consumption of high-GI beverages containing a maltodextrin-sucrose mixture, young men showed a deterioration of glycemic variability (i.e., MAGE). The increase in MAGE evoked by physical inactivity was significantly prevented by the consumption of low-GI beverages with ISO (42).

One plausible explanation for the acute effects of ISO on MAGE is the increased secretion of glucagon-like peptide 1 (GLP-1) and the decreased secretion of glucose-induced insulinotropic peptide (GIP). Besides the insulin-enhancing feature, GLP-1 triggers pancreatic beta-cell proliferation as well as differentiation, which in total leads to the contribution of GLP-1 to limit glucose excursions (43, 44). The GLP-1 increase followed by GIP suppression due to ISO intake has been demonstrated in several intervention studies before (9, 28, 42, 45, 46).

Moreover, we demonstrated a decrease in glycemic variability over time. In agreement, previous studies showed an improvement in different aspects of glucose metabolism following continuous ITF consumption, such as lower postprandial glycemic response to a standardized meal or improved insulin sensitivity (47–50). The beneficial effect on metabolic parameters can be attributed to the selective fermentation of ITF by the gut microbiota. By this, ITF acts as a prebiotic that stimulates the production of bacterial metabolites, like SCFA (23), which are suggested to be key mediators of both, intestinal physiology (e.g., gut integrity, inflammation) and metabolic health (e.g., glucose and energy homeostasis) (51–53). Animals and human studies have furthermore demonstrated an inulin-induced increase in GLP-1 and peptide YY (PYY) which was linked to improved insulin sensitivity, decreased appetite, and reduced body weight gain (47, 49, 54–58). Remarkably, irrespective of the effect achieved by ITF, the difference in glycemic variability between ISO and SAC test days remained even at the end of the intervention. Even though the functional food ingredients, ISO and ITF, work “hand-in-hand” in ameliorating glycemic variability, further research is warranted to elucidate the synergistic nature of these effects.

In terms of the gut microbiome composition, an increase in the relative abundance of *Actinobacteria* as well as *Bifidobacteria* was detected, which is well in line with numerous clinical studies showing the inulin-induced bifidogenic effect (31, 33–35). *Bifidobacteria* have been shown to be beneficial for human health (22, 59). Furthermore, our data confirm previous findings of an increase in butyrate-producing and thus health-promoting *Anearostipes* (31, 60). Taken together, our data confirm the hypothesis of an ITF-induced selective modulation of the gut microbiota composition, also under habitual living conditions and a real-life setting.

Blood glucose regulation is subject to a high interindividual variability (18, 61). Recent data indicate that a person's individual glycemic response might be predicted by anthropometrics or gut microbiota composition (14, 18). We also observed a high interindividual variability in terms of the postprandial glucose response as well as the glycemic variability. With regards to MAGE, we speculate that the improvement in glycemic variability with ISO also varies between individuals and that these individuals differ in their characteristics related to anthropometrics, metabolic- or microbiome-related variables. Remarkably, the assignment of individuals to good responders and low responders according to their magnitude of glycemic fluctuations revealed that the majority showed pronounced improvements while none experienced a disadvantage of the replacement of SAC with ISO.

Microbiome analyses indicated a difference regarding the Firmicutes:Bacteroidetes (F:B) ratio between good responders and low responders. The F:B ratio has been shown earlier to be positively associated with obesity and its comorbidities (19, 62, 63). Our findings demonstrate a trend for a higher F:B ratio in good responders. Due to this finding, we hypothesize that good responders might be characterized by a rather unfavorable metabolic phenotype, e.g., higher body weight, BMI, or insulin resistance. If so, those participants may benefit even more from low-GI carbohydrates and prebiotic fibers.

Our study as a retrospective analysis has several strengths: Data were obtained in a relatively large and heterogenous sample from the general population under habitual lifestyle conditions. The analyses demonstrate the relevant benefits of the functional ingredients tested when integrated into the daily diet. The parameters which were available for this analysis were determined using state-of-the-art microbiome analysis, i.e., 16S rRNA sequencing, as well as increasingly important and very detailed CGM.

We do acknowledge some limitations. Since this was a retrospective analysis, we did not have the possibility to examine the impact on further metabolic parameters like insulinemia, gut hormones, or SCFA. Because of the initial aim of this real-life investigation, there was no control arm for the ITF intervention and potentially relevant confounding factors, such as physical activity or lifestyle changes, cannot be excluded. In addition, the *ad libitum* meals were self-reported as was the adherence to the ingredients by the participants.

## Conclusion

With this retrospective analysis of data from a real-life setting, we demonstrated that the integration of functional food ingredients could exert beneficial health effects and thereby improve diet's quality, as a major determinant for health.

More specifically, we showed that combining prebiotic inulin-type fructans and the low-GI carbohydrate, isomaltulose, in the habitual diet benefits on the postprandial and daylong glycemia, as well as intestinal microbiota. The effects observed were robust even in such a very heterogenous population and not overruled by the habitual diet and usual lifestyle. Still, we detected interindividual differences and data reveal persons who might profit even more from the benefits of the ingredients. Lastly, we provide evidence for a synergistic effect of ISO and ITF on glycemia. Further research in prospective trials is essential to confirm our observations.

## Data Availability Statement

The data presented in the study are deposited in the European Nucleotide Achive repository, accession number PRJEB49980; https://www.ebi.ac.uk/ena/browser/view/PRJEB49980.

## Ethics Statement

Ethical review and approval was not required for the study on human participants in accordance with the local legislation and institutional requirements. The patients/participants provided their written informed consent to participate in this study.

## Author Contributions

CS and TS contributed to the conception and design of the digital nutrition program and performed data collection. LS and ST provided scientific input for the retrospective data analysis. AKü and HB performed the statistical analysis. AKo, MS, and LS wrote the first draft of the manuscript. All authors read and approved the submitted manuscript.

## Funding

Financial support of the modified digital nutrition program was provided by BENEO GmbH (Mannheim, Germany) a member of the Südzucker Group. BENEO GmbH reserves the exclusive right to use the results and data for possible Health Claim requests. HB acknowledges funding by the Deutsche Forschungsgemeinschaft (DFG, German Research Foundation) under Germany's Excellence Strategy—EXC 22167-390884018. The authors declare that this study received funding from BENEO GmbH/Südzucker Group. The funder had the following involvement in the study: provided the test products, LS and ST provided scientific input for the retrospective data analysis, LS contributed to the first draft of the manuscript.

## Conflict of Interest

ST and LS are employees of BENEO/Südzucker Group. TS was employed at Perfood GmbH. TS and CS are co-founders of Perfood GmbH and minority shareholders. AKü obtained the payment for performing the microbiome analysis in this study. The remaining authors declare that the research was conducted in the absence of any commercial or financial relationships that could be construed as a potential conflict of interest.

## Publisher's Note

All claims expressed in this article are solely those of the authors and do not necessarily represent those of their affiliated organizations, or those of the publisher, the editors and the reviewers. Any product that may be evaluated in this article, or claim that may be made by its manufacturer, is not guaranteed or endorsed by the publisher.
