# Supplementary material for: PalatinoseTM (Isomaltulose) and Prebiotic Inulin-Type Fructans Have Beneficial Effects on Glycemic Response and Gut Microbiota Composition in Healthy Volunteers—A Real-Life, Retrospective Study of a Cohort That Participated in a Digital Nutrition Program
Source: Front Nutr. 2022 Mar 7;9:829933. doi: 10.3389/fnut.2022.829933 (PMC8948463; doi:10.3389/fnut.2022.829933)
Supplement: Supplementary file 1 [file Data_Sheet_1.docx]

Supplementary Material

# Supplementary Figures


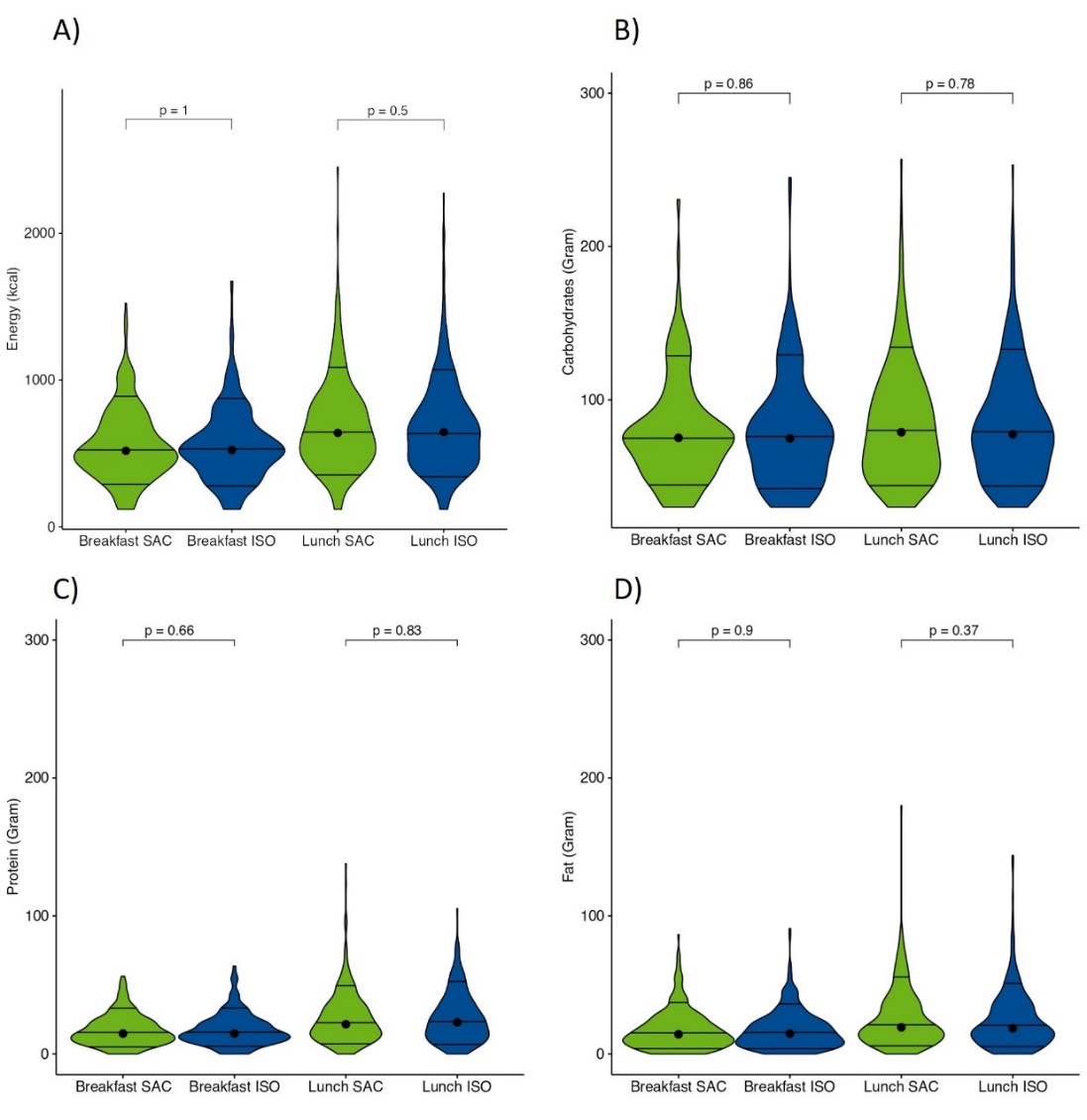


**Supplementary Figure 1.** Energy and macronutrient content of breakfasts and lunches with either SAC (green) or ISO (blue). A) energy intake in kcal, B) carbohydrate intake in gram, C) protein intake in gram, D) fat intake in gram (n=319 and n=318 for breakfast with SAC and ISO, n=301 and n=396 for lunch with SAC and ISO). Significant differences were assessed using the Wilcoxon test: p<0.05.

**
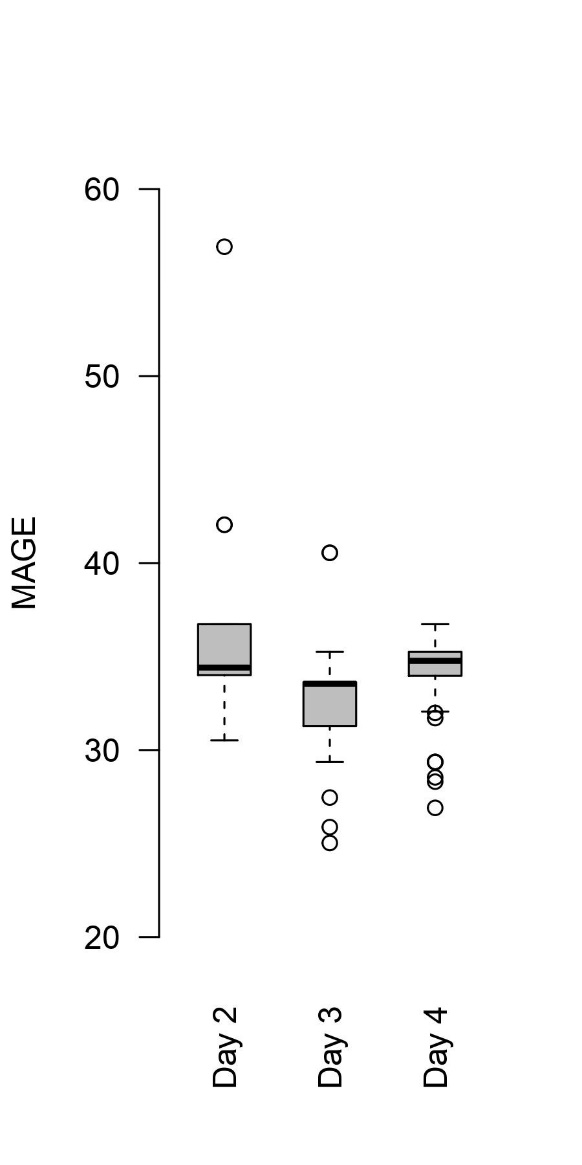
**

**Supplementary Figure 2.** Glycemic variability on test days with SAC (Day 2) and ISO (Day 3) in comparison to the glycemic variability on a control day (Day 4). Day 3 shows a significant reduction compared to day 2 and 4 (Kruskal test p < 0.05 with Dunn test as post-hoc test, p < 0.001).
